# Second hand smoke exposure and the risk of invasive meningococcal disease in children: systematic review and meta-analysis

**DOI:** 10.1186/1471-2458-12-1062

**Published:** 2012-12-10

**Authors:** Rachael L Murray, John Britton, Jo Leonardi-Bee

**Affiliations:** 1 Division of Epidemiology and Public Health, University of Nottingham, Clinical Sciences Building Phase 2, Nottingham City Hospital, Hucknall Road, Nottingham, NG5 1PB, UK

**Keywords:** Second hand smoke, Environmental tobacco smoke, Invasive meningococcal disease, Systematic review, Meta-analysis

## Abstract

**Background:**

Invasive meningococcal disease remains an important cause of serious morbidity and mortality in children and young people. There is a growing body of literature to suggest that exposure to passive smoke may play a role in the development of the disease, therefore we have performed a systematic review to provide a comprehensive estimate of the magnitude of this effect for smoking by any household member, by individual family members, and of maternal smoking before and after birth.

**Methods:**

Four databases (Medline, Embase, PsychINFO and CAB Abstracts database) were searched to identify studies (to June 2012) and reference lists scanned for further studies. Titles, abstracts and full texts were checked for eligibility independently by two authors. Quality of included studies was assessed using the Newcastle-Ottawa Scale. Pooled odds ratios (OR) with 95% confidence intervals (CI) were estimated using random effect models, with heterogeneity quantified using I^2^.

**Results:**

We identified 18 studies which assessed the effects of SHS on the risk of invasive meningococcal disease in children. SHS in the home doubled the risk of invasive meningococcal disease (OR 2.18, 95% CI 1.63 to 2.92, I^2^ = 72%), with some evidence of an exposure-response gradient. The strongest effect was seen in children under 5 years (OR 2.48, 95% CI 1.51 to 4.09, I^2^ = 47%). Maternal smoking significantly increased the risk of invasive meningococcal disease by 3 times during pregnancy (OR 2.93, 95% CI 1.52-5.66) and by 2 times after birth (OR 2.26, 95% CI 1.54-3.31).

**Conclusions:**

SHS exposure, and particularly passive foetal exposure to maternal smoking during pregnancy, significantly increases the risk of childhood invasive meningococcal disease. It is likely that an extra 630 cases of invasive meningococcal disease annually in children under 16 are directly attributable to SHS exposure in UK homes.

## Background

Invasive meningococcal disease is a catastrophic illness that can have devastating effects. It is the most common cause of bacterial meningitis in the UK and Ireland, an important cause of serious morbidity and mortality in children and young adults [1] with nearly half of all cases of laboratory confirmed meningitis in England and Wales being aged under 14 years [2]. With prompt medical intervention the majority of cases make a full recovery, but around 16% are left with at least one major adverse outcome, such as severe intellectual disability, epilepsy, spasticity or deafness, whilst nearly 5% of cases are fatal [3]. At 7 and 12 years after infection, meningitis survivors demonstrate significantly lower IQ scores and higher incidences of neurological or behavioural disorders, and particularly those who developed meningitis during infancy and experienced neurological complications at the time of the illness [4,5].

A growing body of literature suggests that exposure to second hand smoke (SHS) may play a role in the development of meningococcal disease. A recent meta-analysis limited to 16 large studies estimated this increase in risk at a relative odds of 2.30 (95% CI 1.74 to 3.06) among children with one or more parent who smoked [6]. Another recent systematic review found exposure to SHS was associated with a two-fold increased risk of invasive meningococcal disease in those aged between 1 month and 19 years old [7], but this study did not explore the effects of smoking by different family members, or the effects of prenatal smoke exposure.

We have therefore carried out a full systematic review and meta-analysis of all available epidemiological evidence to provide a comprehensive estimate of the effect of SHS by different family members, and of pre- and post-natal maternal smoking, on the risk of invasive meningococcal disease in different stages of childhood.

## Methods

### Systematic review methods

We identified all comparative epidemiological studies (case–control, cross-sectional, cohort designs) assessing the association between SHS exposure and the risk of invasive meningococcal disease in children (aged < 18 years) through a comprehensive search of three electronic databases (Medline, Embase, and PsychINFO, searched to June 2012), by scanning reference lists of the included studies, and using the CAB Abstracts database (June 2012) to identify relevant conference abstracts. Case reports, case series and grey data were not included. The following search terms were used to identify studies (where ‘mp’ indicates the text was searched for in the abstract, titles, original titles, broad terms, and heading words; ‘/’ indicates MeSH terms, ‘exp’ indicates explosion of MeSH terms): tobacco.mp; cigarette smoke.mp; smoker.mp; smoking.mp; cigar.mp; exp tobacco/; exp tobacco dependence/; exp tobacco smoke/; exp cigarette smoke/; exp cigarette smoking/; exp smoking/; exp smoke/; exp "smoking and smoking related phenomena"/; exp adolescent smoking/; exp parental smoking/; exp passive smoking/; exp smoking habit/; exp smoking cessation/; exp Crowding/; exp tobacco smoke pollution/; second hand smoke.mp; meningitis.mp; septicaemia.mp; meninges.mp; bacterial meningitis.mp; viral meningitis.mp; fungal meningitis.mp; cryptococcal meningitis.mp; exp meningioma/; exp meningism/; exp meningitis/; exp central nervous system infection/; exp meninx disorder/; exp nervous system inflammation/; exp arachnoiditis/; exp aseptic meningitis/; exp bacterial meningitis/; exp epidemic meningitis/; exp fungal meningitis/; exp group b streptococcal meningitis/; exp haemophilus meningitis/; exp lymphocytic choriomeningitis/; exp meningoencephalitis/; exp pneumococcal meningitis/; exp primary amebic meningoencephalitis/; exp subdural empyema/; exp tuberculous meningitis/; exp virus meningitis/; exp vogt koyanagi syndrome/; exp septicemia/; exp sepsis/; exp candida meningitis/; exp cryptococcal meningitis/; exp meningitis, bacterial/; exp meningitis, meningococcal/; exp meningococcal infections/; exp meningitis, pneumococcal/; exp pneumococcal infections/; exp Streptococcus pneumoniae/; exp meningitis, haemophilus/; exp haemophilus infections/; meningitis, viral/; meningitis, aseptic/; virus diseases/; neisseria/; neisseria meningitidis/; neisseria meningitidis, serogroup a/; neisseria meningitidis, serogroup b/; neisseria meningitidis, serogroup c/; neisseria meningitidis, serogroup w-135/; neisseria meningitidis, serogroup y/. We imposed no language restrictions.

### Exposure measures

We included all sources of SHS exposure (parental, household, carer, other family members) measured by either self-report (typically by questionnaire) or biochemical markers of exposure such as cotinine in saliva. Exposures were classified as either in-utero, postnatal, infant, or childhood exposure. Studies of the effects of active smoking were excluded.

### Outcome measures

We included all studies with invasive meningococcal disease diagnosed clinically, and/or by laboratory confirmation through identification of pathogens in blood or CSF, as the outcome. We excluded studies of meningococcal carriage, and studies in which invasive meningococcal disease outcomes could not be clearly extracted from a broader group of diagnoses.

### Study selection

Titles and abstracts identified from the searches were checked for eligibility independently by two authors (either RLM and MM, or JLB and MM). Studies deemed not to be relevant were excluded at each stage. The full text of potentially eligible papers was sought and also checked for eligibility independently by two authors (either RLM and MM, or JLB and MM). Disagreements were resolved through discussion with a third author. Data from the included studies relating to a study design, participants, outcome measures and results were extracted using a piloted data extraction form independently by two authors (either RLM and MM, or JLB and MM). Any paper not published in English was translated at the relevant stage of screening, with data extracted as per English language papers.

### Assessment of methodological quality

Studies deemed eligible for inclusion in the systematic review were scored for methodological quality using the Newcastle-Ottawa Assessment Scale [8]. The scale consists of three categories relating to selection, comparability and ascertainment of exposure (for case–control and cross-sectional studies) or ascertainment of outcome (for cohort studies), with a maximum score of 9 being awarded for the highest quality studies. This process was performed independently by two authors (either RM and MM, or JLB and MM), with disagreements resolved through discussion with a third author. A score of six or more was taken to distinguish higher from lower quality studies.

### Statistical analysis

Estimates of effect were extracted from the papers and analysed to estimate measures of effect either using unadjusted (crude) odds ratios (OR), or where possible, using adjusted ORs; with their associated 95% confidence intervals (CI). Meta-analysis was used to estimate the effects of SHS exposure and the risk of invasive meningococcal disease using random effects models, due to anticipated high levels of heterogeneity between the included studies. Separate analyses were carried out for each exposure measure (in-utero and childhood exposure) where possible. For studies which solely reported categories of cigarette consumption, we used the highest category of cigarette consumption for the analyses. Heterogeneity was quantified using recognised methods (I^2^) [9], and where high levels of heterogeneity (I^2^ > 50%) were detected between the studies we performed subgroup and sensitivity analyses to explore the effects age of the participants (<5 versus ≤18 years of age), ascertainment of disease (laboratory confirmed cases only versus case definition including non confirmed cases), and methodological quality of the studies (score <6 versus ≤6). Where extreme levels of heterogeneity were detected between the studies (I^2^ > 80%), we did not perform an overall meta-analysis.

Small study bias (publication bias) was assessed visually using a funnel plot and Eggers test for Asymmetry [10]. The effect of publication bias was assessed using the ‘Tim and Fill’ procedure [11], by which the pooled OR and 95% CI are re-calculated after imputation of the results of hypothetically missing studies which would be needed to minimise the effect of publication bias. Statistical analysis was performed using Review Manager 5 (Review Manager (RevMan) [Computer program]. Version 5.0. Copenhagen: The Nordic Cochrane Centre, The Cochrane Collaboration, 2008), and STATA version 11 MP (StataCorp LP, 4905 Lakeway Drive, College Station, TX, USA). P values <0.05 were taken as statistically significant. We adhered to the MOOSE guidelines throughout the review process [12].

### Population attributable fraction estimation

We estimated the proportion of children in England (aged four to 15 years) who live in a household in which at least one person smokes using data from the Health Survey for England [6], and the formula *p(OR-1)/[p(OR-1) + 1],* in which *p* is the proportion of the cohort exposed to SHS (defined as the proportion of children who did not live in a smoke-free home, where a smoke-free home was defined as living in a home without regular smoking indoors), and *OR* the odds ratio for invasive meningococcal disease in children where a member of the household smokes, to estimate the proportion of children with invasive meningococcal disease attributable to household smoking exposure. We then used national invasive meningococcal disease incidence data for the UK [6] to estimate the number of disease episodes generated as a result of household SHS exposure.

## Results

### Overview of included studies

**Figure 1:**
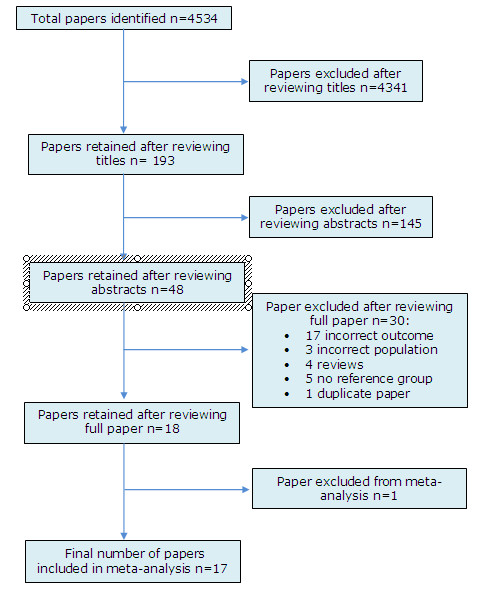
Flow diagram of included and excluded studies.

**Figure 2:**
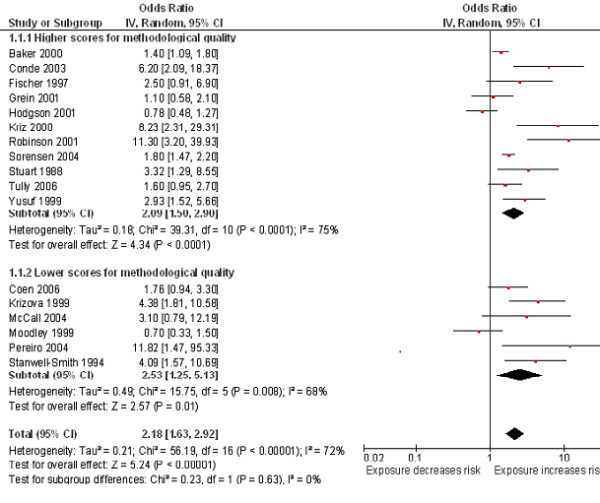
**Smoking by any smoker in the household and the risk of invasive meningococcal disease: subgroup analysis by methodological quality.** Squares indicate the odds ratio and bars represent 95% confidence intervals (CI). Odds ratios more than one indicate smoke exposure in the household increases the risk of invasive meningococcal disease in children.

Of a total of 4534 papers identified from the searches, 193 titles were deemed to be potentially eligible for inclusion. After checking the abstracts of these 193 studies we identified 48 potentially eligible papers. The full texts of these papers were reviewed, and 18 deemed eligible for inclusion in the systematic review (Figure 1, Table 1). The 30 full text papers that were excluded comprised 10 that investigated meningococcal carriage rather than invasive meningococcal disease [13-22], two in which invasive meningococcal disease could not be distinguished from a wider group of disease outcomes [23,24], four that looked only at Haemophilus influenza type B (HiB) and Pneumococcal infections [25-28], one that studied bacterial sepsis [29], three that focussed only on adult populations [30-32], four that were reviews [33-36], five with no reference group [37-41], and one duplicate report of a study included in the review [42] (Figure 2).

**Table 1:** Characteristics of included studies

**Author & Year**	**Setting**	**Design and study population**	**Exposure**	**Ascertainment of disease**	**NOS**
Baker 2000 [51]	New-Zealand	Case–control: 202 cases, 313 controls	One or more smokers in household	Laboratory confirmed and probable	6
Coen 2006 [52]	England	Case–control: 144 cases, 144 controls	Exposure to smokers, exposure to smoke.	Laboratory confirmed and probable	5
Conde 2003 [45]	Portugal	Case–control: 47 cases, 51 controls	Maternal smoking	Laboratory confirmed	6
Fischer 1997 [46]	State of Washington, USA	Case–control: 129 cases, 274 controls	Maternal smoking, passive tobacco smoke	Laboratory confirmed	7
Grein 2001 [47]	Republic of Ireland	Case–control: 87 cases, 267 controls	Household smoking	Laboratory confirmed	8
Haneberg 1983 [53]	Norway	Case–control: 115 cases, 61 patient controls, 293 population controls	Heavy/moderate smoke exposure	Laboratory confirmed and probable	4
Hodgson 2001 [54]	Kassena-Nankana district, Ghana.	Case–control: 505 cases, 505 controls.	Paternal smoking	Laboratory confirmed and probable	8
Kriz 2000 [48]	Czech republic	Case control: 68 cases, 135 controls	Maternal smoking, Paternal smoking, Maternal smoking only, Paternal smoking only, Both parents smoking, Smoking at home per 20 cigarettes a day	Laboratory confirmed	7
Krizova 1999 [55]	Czech republic	Case control: 107 cases, 211 controls	Maternal smoking, Paternal smoking, another member of family	Laboratory confirmed and probable	5
McCall 2004 [49]	Area covered by the SPHUN, Queensland, Australia	Case control: 62 cases, 79 controls	Passive tobacco smoke exposure, carer smoking.	Laboratory confirmed	4
Moodley 1999 [56]	Cape town metropolitan region, South Africa.	Case control: 70 cases, 210 controls	More than 2 Smokers per household, Main caregiver smokes	Laboratory confirmed and probable	3
Pereiro 2004 [57]	Valencia, Spain	Case control: 181 cases, 243 controls.	Under 15s: No. of smokers, Maternal smoking, Paternal smoking, Other smoking, No of cigarettes smoked by other at home <10, 10 – 20, 20>. Under 5s: No of cigarettes smoked; 10 to 29,30 to 59.	Laboratory confirmed and probable	5
Robinson 2001 [58]	Victoria, Australia	Case control: 47 cases, 94 controls	Smoker amongst intimate contact.	Laboratory confirmed and probable	7
Sorensen 2004 [59]	Denmark	Case control: 462 cases, 9240 controls.	Maternal smoking	Laboratory confirmed and probable	7
Stanwell-Smith 1994 [50]	West England	Case control: 74 cases, 232 controls	Any household smoker, Smoking at home, Smoking on visits, Cigarettes smoked per day in home;1-9, 10-19, 20-29, 30 or more. Number of smokers in the household; None, One, Two, Three or more.	Laboratory confirmed	5
Stuart 1988 [60]	England	Case control: 105 cases, 105 controls.	Other smokers in the household	Laboratory confirmed and probable	6
Tully 2006 [43]	England	Cohort: 144 cases, 144 controls	Multiple close contacts who smoke	Laboratory confirmed	9
Yusuf 1999 [44]	Atlanta, USA	Cohort: 283291 people, including 55 cases.	Mother smoked during pregnancy	Laboratory confirmed	8

*NOS* Newcastle-Ottawa Scale for assessing methodological quality of studies.

The majority of the included studies used case–control designs, and two were cohort studies [43,44]. Cases in the included studies were defined in different ways, with eight studies using only laboratory confirmed diagnosis [43-50] , with one taking cases identified by death certificates [44]. In the remaining 10 studies, case definition allowed for both laboratory confirmed diagnoses and non-confirmed (probable) diagnoses, through clinical diagnosis of symptoms [51-60].

### Methodological quality of studies and Publication Bias

The methodological quality scores for the included studies ranged from 3 to 9, with a median 6; 11 studies (61%) were deemed to be of a high quality (≥6). The median scores for the three categories were 3 for selection, 2 for comparability and 1.5 for ascertainment of exposure/outcome. Lower quality scores tended to arise from methods of selection and ascertainment of exposure/outcome.

### Passive smoke exposure in the household

All of the included studies assessed exposure to SHS using questionnaires. A pooled analysis found exposure to SHS by any smoker in the household more than doubled the risk of invasive meningococcal disease (OR 2.18, 95% CI 1.63 to 2.92, I^2^ = 72%, 17 studies; Figure 2). Seventeen of the included papers were used in the meta-analysis, the exception being one that did not provide effect estimates; however, this study found a significant increase in the risk of meningitis in children <12 years who were exposed to moderate or heavy amounts of passive smoke (P < 0.001) [53].

**Figure 3:**
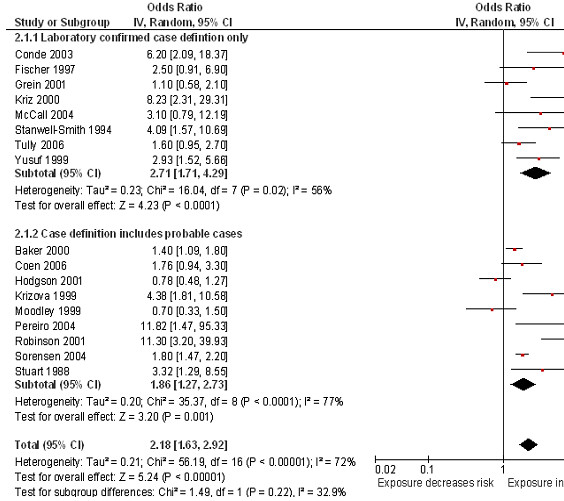
**Smoking by any smoker in the household and the risk of invasive meningococcal disease: subgroup analysis by case definition.** Squares indicate the odds ratio and bars represent 95% confidence intervals (CI). Odds ratios more than one indicate smoke exposure in the household increases the risk of invasive meningococcal disease in children.

**Figure 4:**
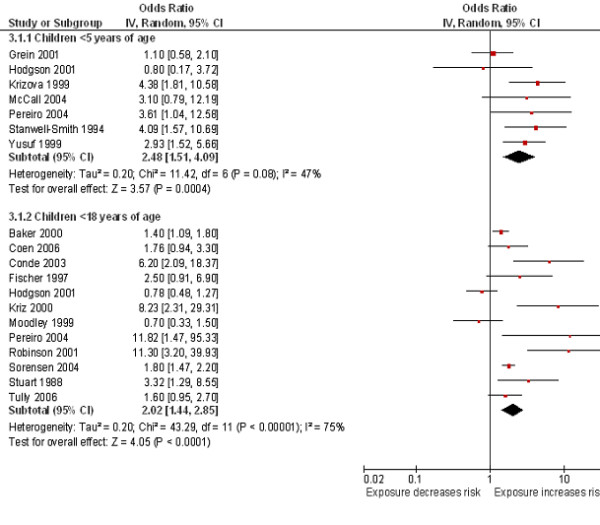
**Smoking by any smoker in the household and the risk of invasive meningococcal disease: subgroup analysis by age of study population.** Squares indicate the odds ratio and bars represent 95% confidence intervals (CI). Odds ratios more than one indicate smoke exposure in the household increases the risk of invasive meningococcal disease in children.

Subgroup analysis based on methodological quality found higher pooled estimates for lower quality studies (OR 2.53, 95% CI 1.25 to 5.13; I^2^ = 68%) than higher quality (OR 2.09, 95% CI 1.50 to 2.90; I^2^ = 75%) studies (Figure 2). A subgroup analysis based on case definition found the estimates in those studies that used laboratory confirmed cases only were more homogeneous (OR 2.71, 95% CI 1.71 to 4.29, I^2^ = 56%) compared to studies which included non-confirmed (probable) cases (OR 1.86, 95% CI 1.27 to 2.73, I^2^ = 77%) (Figure 3). Stronger magnitudes of effect were seen for the association between SHS exposure in the household and the risk of invasive meningococcal disease in children under 5 years (OR 2.48, 95% CI 1.51 to 4.09, I^2^ = 47%) compared to the association seen in children <18 years (OR 2.02, 95% CI 1.44 to 2.85, I^2^ = 75%) (Figure 4).

### Maternal or paternal smoking

**Figure 5:**
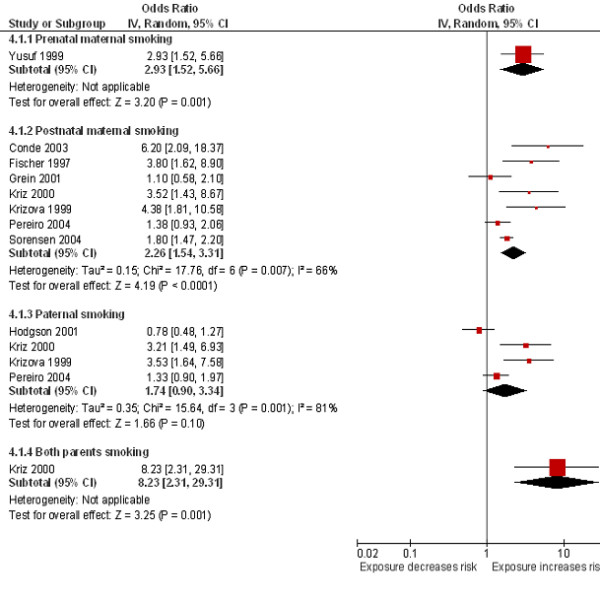
**Maternal and paternal smoking and the risk of invasive meningococcal disease.** Squares indicate the odds ratio and bars represent 95% confidence intervals (CI). Odds ratios more than one indicate maternal and paternal smoke exposure increases the risk of invasive meningococcal disease in children.

One paper that assessed smoking in the mother during pregnancy found more than a doubling of the risk of invasive meningococcal disease (OR 2.93, 95% CI 1.52 to 5.66; Figure 5). Exposure to maternal smoking after birth was also found to double the risk of invasive meningococcal disease (OR 2.26, 95% CI 1.54 to 3.31, I^2^ = 66%, 7 studies; Figure 5). Extreme levels of heterogeneity were detected between the studies which assessed the effect of exposure to paternal smoking on the risk of invasive meningococcal disease (I^2^ = 81%; Figure 5); therefore, the results for these four studies are presented narratively. Two of the studies demonstrated significantly increased risks of invasive meningococcal disease associated with exposure to parental smoking (OR 3.21, 95% CI 1.49 to 6.93 [48]; OR 3.53, 95% CI 1.64 to 7.58 [55]; however, the remaining studies either showed a non-significant increase (OR 1.33, 95% CI 0.90 to 1.97 [57] or a non-significant decrease (OR 0.78, 95% CI 0.48 to 1.27 [54]. One study that assessed exposure to both parents smoking found an eight-fold increase in the risk of invasive meningococcal disease (OR 8.23, 95% CI 2.31 to 29.31; Figure 5).

### Publication bias

**Figure 6:**
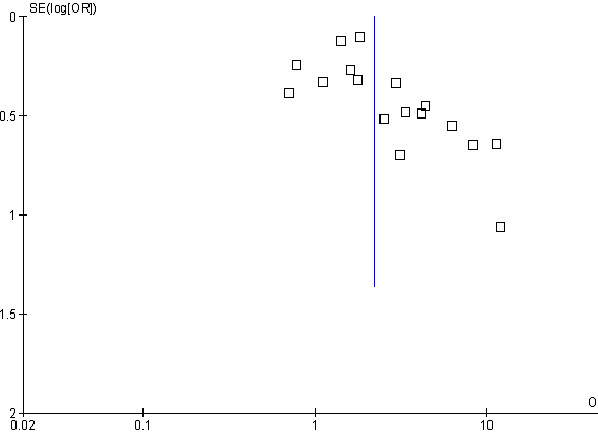
Smoking by any smoker in the household and the risk of invasive meningococcal disease: funnel plot.

There was some evidence of small study bias (publication bias) identified in the funnel plot (Figure 6) and Egger’s test (p = 0.033) for the association between exposure to household smoke and the risk of invasive meningococcal disease (17 studies included). The ‘Trim and Fill’ procedure identified six studies would be needed to minimise the effect of publication bias. The ORs and 95% CI for the six hypothetical studies were imputed into the meta-analysis, which resulted in a bias-corrected OR of 1.59 (95% CI 1.17 to 2.15, p = 0.003; 23 studies).

### Population attributable fraction

Health survey for England data indicate that in 2007, around 22% of children aged from four to 15 years lived in a household in which someone smokes (defined as the proportion of children who did not live in a smoke-free home, where a smoke-free home was defined as living in a home without regular smoking indoors) [6]. Using the pooled odds ratio for household smoking from our meta-analysis (2.18) as the estimated relative risk of developing invasive meningococcal disease, the proportion of children developing invasive meningococcal disease likely to be attributable to exposure to smoking in the home is estimated at 20.6%. In 2008 there were approximately 3,070 events of invasive meningococcal disease in children under the age of 16 years in the UK [6]. A 20.6% attributable fraction translates into approximately 630 new cases of invasive meningococcal disease each year arising from exposure to smoking in the home in the UK. Allowing for the effects of publication bias using the bias-corrected odds ratio for household smoking (1.59) results in a bias-corrected population attributable fraction of 11.5%, which translates to an additional 350 cases of invasive meningococcal disease per year arising from exposure to smoking in the home.

## Discussion

This systematic review and meta-analysis of all available epidemiological evidence of the effect of SHS exposure on the clinical manifestation of invasive meningococcal disease confirms that exposure to SHS in the household more than doubles the risk of invasive meningococcal disease, that this finding is consistent across both higher and lower quality studies, and is stronger for laboratory confirmed than clinically diagnosed outcomes. The effect appears to be particularly strong in households in which both parents smoke, and with prenatal or postnatal maternal smoking. The effects were strongest in children aged under five years. We estimate that around 630 UK cases of invasive meningococcal disease in children under 16 years are currently attributable to maternal or paternal smoking. With an estimated 5% mortality this translates into about 30 deaths per year.

### Strengths and limitations

The data included in this review were included after comprehensive literature searches and hand searching of previous reviews and reference lists of published articles. Our findings are therefore likely to be representative of the true effect of SHS exposure on the development of invasive meningococcal disease. This review built on a recent meta-analysis that we performed as part of a broader review of the effects of passive smoking in children, for a Royal College of Physicians report [6]. However, the meta-analysis was limited as it included clinically heterogeneous studies of meningococcal and other bacterial meningitis, and also included studies assessing the effects on meningococcal carriage. Furthermore, the review failed to investigate reasons for heterogeneity between the studies using subgroup analysis. In the current review, we have presented a more comprehensive systematic review which advances the findings previously published by addressing the limitations above. Further to the review published in 2010 [7], we were able to include an additional three eligible studies [44,45,55] which were previously not identified or included. Two of the included studies used very similar populations based on a surveillance program with data collection over the same period of time [48,55]; however, both were included in the analyses as they contributed to different subgroup categories, therefore there was insufficient evidence to ascertain whether they were mutually exclusive. We imposed no language restrictions on the search, which allowed us to include all articles of interest. The current study was limited by an inability to adjust for a full range of confounders in the analyses due to large variations in the published papers. Higher quality studies tended to adjust for age, overcrowding and socio-economic status but this was not always the case, particularly in lower quality studies. Factors such as hygiene and exposure to other sources of smoke, such as from cooking (as found by Hodgson et al. 2001 [54]), were not commonly adjusted for and may have affected the results gained. There was also some evidence of publication bias towards smaller studies showing either a protective effect or no effect; however, the bias-corrected pooled estimate still demonstrated a strong and significant increase in the odds of invasive meningococcal disease associated with exposure to SHS. Varied definitions of SHS were used within the included studies and ranged from only including smoking from particular household members, for example based solely on exposure to smoking from the mother; through to assessing smoke exposure from more than one source, for example based on exposure to smoking from any household family members, visitors to the household, and exposure at day care settings. It would have been interesting to separate ‘exposure to smokers' from 'exposure to smoking' to investigate any differential effects, however, meaningful subgroup comparisons were not possible due to the numerous ways that the included studies defined passive smoke exposure, as indicated in Table 1. A further limitation of the review relates to the inability to assess whether there was evidence of effect modification, for example by crowded households, due to the studies not presenting their data in suitable formats to enable this.

This review has demonstrated that the increase in risk of invasive meningococcal disease is primarily due to smoking by the mother, either during pregnancy or post-natally. It is a difficult to disentangle the independent effects of smoking during pregnancy from that in the postnatal period due to the high concordance of smoking by the mother in the two periods; however smoking in pregnancy is known to increase the risk of other various childhood infections, for example lower respiratory infection [61]. Only one study included in this review assessed the effect of smoking in pregnancy on the risk of invasive meningococcal disease [44], which demonstrated a substantial increase in risk; however, further well-conducted studies are needed to describe this association conclusively, and to assess the effect of SHS smoke exposure during different stages of pregnancy.

Sir Austin Bradford Hill’s postulates to review whether there is sufficient evidence in support of a causative role of SHS exposure on invasive meningococcal disease [62]. The meta-analysis conducted in this review demonstrated a *consistent,* marked *strength in association* between SHS exposure and invasive meningococcal disease; however, the *specificity criterion* is unlikely to be met, since invasive meningococcal disease is known to be caused by factors other than exposure to SHS. In terms of *temporal sequence*, we only identified two cohort studies of passive smoking and invasive meningococcal disease [43,44]; both of the studies were judged to be of high quality and both demonstrated clear marked increases. There was some evidence of a *biological gradient* where stronger magnitudes of effect were seen in those exposed to higher numbers of cigarettes smoked in the home per day [50,57], specifically where no effect was seen in those exposed to less than 10 cigarettes per day, but a doubling in risk of invasive meningococcal disease was seen in those exposed to moderate amounts of cigarettes per day, and a 3 to 4 fold increase in those heavily exposed to cigarette smoke per day. In terms of *biological plausibility* and *coherence* with what is already known, the possible mechanisms for how SHS exposure may induce invasive meningococcal disease may be related to the effects of tobacco acting systemically reducing the defences of the nasopharyngeal mucosa against potential pathogens [46,50]. However, current knowledge also suggests higher carriage rates in smoking parents [63], thus exposure to smoke in childhood may increase the exposure to the pathogen. Very little research has been performed to support the *experimental evidence* criterion by, for example, assessing the effects of smoking cessation in homes of children exposed to parental smoke on the reduced risk of invasive meningococcal disease, however, in terms of *analogous evidence*; there is growing evidence to suggest the causal effect of SHS exposure on other childhood infections. Therefore there seems to be some evidence to support SHS as a causative factor of invasive meningococcal disease.

The results of this study are limited to presentations of confirmed or clinically probable invasive meningococcal disease as these are the outcomes directly relevant to estimating the effect of SHS on disease risk. We did not explore the effects of SHS on carriage rate, though such studies would be of value to determine whether smoking increases the risk of disease through increased carriage, or increased progression to disease among those carrying the relevant pathogens.

## Conclusion

Our results demonstrate a further means by which passive smoking presents a significant risk to the health and wellbeing of young children. Interventions to prevent SHS exposure to cigarette smoke from parents and other household members therefore remain an urgent priority.

## Competing interests

The authors declare that they have no competing interests.

## Authors’ contributions

RLM reviewed the full text articles, extracted data and wrote the initial draft of the manuscript. JLB conducted the literature search, reviewed titles, abstracts and full text articles, extracted data, conducted the statistical analysis and provided critical revision of the manuscript. JB contributed to the critical revision of the manuscript. All authors read and approved the final manuscript.

## Pre-publication history

The pre-publication history for this paper can be accessed here:

http://www.biomedcentral.com/1471-2458/12/1062/prepub
